# Spinal Concussion

**Published:** 1893-09

**Authors:** 


### SPINAL CONCUSSION.

In reality spinal concussion is a temporary condition and ordinarily of brief duration, lasting a few hours, or days at most.

Authors have erroneously considered under this heading many of its consequences, such as the psychoses and secondary inflammation of the cord and its membranes.

For the sake of convenience only, and in order to be in fashion, I have included under "concussion" the primary shock as well as the subsequent sequences after spinal injuries.

Spinal concussion, as thus considered, has no demonstrable pathology attached to it, and all secondary inflammations should be designated as myelitic or some variety of meningeal inflammation, and should be treated as such.

In this disease the injury is thrust upon the sympathetic system of nerves through the perceptive centers of the brain, and not through any inflammatory process of the cord ; so that persons asleep or intoxicated at the time of an accident, and those whose attention is riveted upon some grossly injured member of the body, are always the lightest sufferers after such accidents.

In its nature, spinal concussion is a true hypochondriasis, and is kept alive by morbid suggestions and evil forebodings from self and others, as well as perpetuated by a lack of self-confidence and a neglect of proper exercise, both physical and mental.

Being a disease with few if any objective symptoms, it is often the avenue adopted by malingerers to claim pecuniary reward for home-manufactured injuries.

Absolute rest after injury, and mental diversion with light bodily exercise (especially remunerative exercise) in the secondary stages, are the best means known for averting chronicity and warding off incurableness in spinal concussion.

Where the foregoing methods are properly carried out, the prognosis in this disorder, both as to life and future usefulness, will be very good.—*Dr. Wilkinson in Medical Age, July 25, 1893.*

